# Contrasting alterations to synaptic and intrinsic properties in upper-cervical superficial dorsal horn neurons following acute neck muscle inflammation

**DOI:** 10.1186/1744-8069-10-25

**Published:** 2014-04-12

**Authors:** Belinda M Harris, David I Hughes, Philip S Bolton, Melissa A Tadros, Robert J Callister, Brett A Graham

**Affiliations:** 1 School of Biomedical Sciences and Pharmacy, Faculty of Health and Medicine, The University of Newcastle and Hunter Medical Research Institute, Room 411 Medical Sciences Building, University Drive, Newcastle, NSW 2308, Australia; 2 Institute of Neuroscience & Psychology, College of Medical, Veterinary & Life Sciences, University of Glasgow, Glasgow, UK

**Keywords:** Mice, A-current, EPSC, Carrageenan, Action potential

## Abstract

**Background:**

Acute and chronic pain in axial structures, like the back and neck, are difficult to treat, and have incidence as high as 15%. Surprisingly, most preclinical work on pain mechanisms focuses on cutaneous structures in the limbs and animal models of axial pain are not widely available. Accordingly, we developed a mouse model of acute cervical muscle inflammation and assessed the functional properties of superficial dorsal horn (SDH) neurons.

**Results:**

Male C57/Bl6 mice (P24-P40) were deeply anaesthetised (urethane 2.2 g/kg i.p) and the rectus capitis major muscle (RCM) injected with 40 μl of 2% carrageenan. Sham animals received vehicle injection and controls remained anaesthetised for 2 hrs. Mice in each group were sacrificed at 2 hrs for analysis. c-Fos staining was used to determine the location of activated neurons. c-Fos labelling in carrageenan-injected mice was concentrated within ipsilateral (87% and 63% of labelled neurons in C1 and C2 segments, respectively) and contralateral laminae I - II with some expression in lateral lamina V. c-Fos expression remained below detectable levels in control and sham animals. In additional experiments, whole cell recordings were obtained from visualised SDH neurons in transverse slices in the ipsilateral C1 and C2 spinal segments. Resting membrane potential and input resistance were not altered. Mean spontaneous EPSC amplitude was reduced by ~20% in neurons from carrageenan-injected mice versus control and sham animals (20.63 ± 1.05 vs. 24.64 ± 0.91 and 25.87 ± 1.32 pA, respectively). The amplitude (238 ± 33 vs. 494 ± 96 and 593 ± 167 pA) and inactivation time constant (12.9 ± 1.5 vs. 22.1 ± 3.6 and 15.3 ± 1.4 ms) of the rapid A type potassium current (I_Ar_), the dominant subthreshold current in SDH neurons, were reduced in carrageenan-injected mice.

**Conclusions:**

Excitatory synaptic drive onto, and important intrinsic properties (i.e., I_Ar_) within SDH neurons are reduced two hours after acute muscle inflammation. We propose this time point represents an important transition period between peripheral and central sensitisation with reduced excitatory drive providing an initial neuroprotective mechanism during the early stages of the progression towards central sensitisation.

## Introduction

Acute inflammation in peripheral structures, such as muscle, results in nociceptor activation and pain. Unfortunately, pain sometimes outlasts the initial inflammatory insult and persists in a more chronic form. Long-term changes in components of the pain neuroaxis such as altered synapses and neuronal properties are thought to underlie this process [1]. Understanding the mechanisms involved in the progression from the acute to chronic pain state, however, remains a major challenge in pain neurobiology.

Regardless of the site involved in chronic pain states, the central processing of nociceptive signals begins in the superficial dorsal horn (SDH; laminae I-II) of the spinal cord [2]. Here, the passage of information to be relayed from primary afferent fibres to higher brain regions can be gated by synaptic inputs from local segmental interneurons and descending brainstem centres. Such a process greatly influences whether nociceptor activation can ultimately be perceived as pain [3]. Since the initial discovery that the electrophysiological properties of neurons in the lumbar spinal cord could be altered after repeated noxious peripheral input [4], plasticity in spinal cord pain circuits, termed central sensitisation, has been considered a key element in the development of chronic pain [5,6].

Surprisingly, most of our understanding of the spinal mechanisms underlying plasticity in pain circuits comes from studies on the rodent hindlimb following nerve injury or inflammation, even though there is both clinical and pre-clinical evidence suggesting that such mechanisms may differ for axial and limb pain [7]. Patients with neck pain often complain of a range of symptoms not obviously associated with damage to neck structures. These include dizziness, visual disturbances, general weakness, numbness or parathesis, cutaneous hyperalgesia, as well as psychological symptoms such as disturbances in concentration and memory [8]. These signs and symptoms are not normally associated with limb pain where sensory disturbances tend to be more localised [9]. At the cellular level we know that upper cervical SDH neurons receive input from a unique combination of tissues including cutaneous and deep structures in the neck, head and cranial vault [10,11]. This afferent convergence presumably plays an important role in the complex presentation of pain originating in neck tissues [12]. Together these data suggest the mechanisms underlying processing of nociceptive signals originating in neck structures differ from those in the limbs, and may be important for the development of neck pain and its symptoms.

In order to begin to understand the spinal mechanisms that underlie the development of chronic neck pain, we assessed the impact of acute muscle inflammation on the synaptic and intrinsic properties of SDH neurons in the mouse upper cervical spinal cord. After two hours of acute inflammation of the rectus capitus major (RCM) muscle we find excitatory synaptic drive to SDH neurons and the rapid A-type potassium current are reduced in SDH neurons. We hypothesise that two hours post inflammation is an important epoch during the cascade of events where aberrant nociceptive signaling transitions from a peripheral to central locus.

## Methods

The University of Newcastle Animal Care & Ethics Committee approved all experimental procedures. Male C57/Bl6 mice (P24 - P40) were deeply anaesthetised with urethane (2.2 g/kg i.p) and underwent the following treatments: control animals remained anaesthetised for 2 hrs; sham animals received a unilateral injection of 40 μl phosphate-buffered saline (PBS, pH 7.4) via a 26 gauge needle into the RCM muscle; experimental animals received an injection of 2% carrageenan (in phosphate buffered saline - PBS) into the RCM. This muscle is particularly large in rodents and runs from the spinous process of C2 to the skull. It is easily located by palpating the external occipital protuberance (inion) and the spinous process of the C7 vertebrae. The location of the C1 spinous process in the mouse is one-fifth the distance between the inion and the C7 spinous process. The needle was inserted just lateral to the midline at the level of C1 (angled ~45° to the horizontal). The needle was inserted until it reached the occiput and withdrawn so the tip remained in the RCM muscle belly. Mice were maintained under anaesthetic for 2 hours prior to preparation for immunohistochemistry or electrophysiology.

### Immunohistochemistry

After 2 hours of urethane anaesthesia animals were further injected with Ketamine (100 mg/kg), and perfused through the ascending aorta with PBS (20 ml), followed by 20 ml of 4% paraformaldehyde in 0.1 M phosphate buffer (PB; pH 7.4). The cervical spinal cord (obex - C5) was removed and post-fixed in 4% paraformaldehyde (2 hours) prior to washing with PB and cryoprotection in PB containing 30% sucrose at 4°C for 48 hrs. Serial transverse sections (60 μm) were cut and every 5th section was processed for Fos-like immunoreactivity.

Sections from carrageenan-injected (n = 3), sham 128 (PBS; n = 3), and control (uninjected; n = 4) animals were incubated in 50% ethanol for 30 minutes to enhance antibody penetration and then incubated in 0.03% hydrogen peroxide in PB for 20 minutes to block endogenous peroxide activity. Sections were then incubated for 72 hours with a polyclonal goat anti-fos antibody (diluted 1:1000 in PBS; Santa Cruz Biotechnology, Santa Cruz, CA, USA), followed by a biotinylated donkey anti-goat IgG secondary antibody (1:500; Jackson Immunoresearch, West Grove, PA, USA) for an additional 24 hours. Sections were then incubated in ExtrAvidin-peroxidase (diluted 1:1000. Sigma-Aldrich; Cat no. E2886) and peroxidase labeling visualised using 3′3′-diamino-benzadine (DAB) as a chromagen. All antibodies and the peroxidase-labelled ExtrAvidin were diluted in PBS containing 0.3% Triton X-100. Sections were then dehydrated, cleared and mounted in serial order on slides. The relative position of all c-Fos positive cells within the C1 and C2 segments were plotted onto templates of the appropriate spinal segment (adapted from [13]) using Adobe Illustrator CS4 (Adobe Systems Inc., San Jose, CA, USA). Mean number of c-Fos positive cells per spinal segment was calculated from these data.

### Electrophysiology

Sham (n = 15 mice) and carrageenan injected mice (n = 10) were decapitated as close as possible to the thorax while still under urethane anaesthesia. Control animals (uninjected; n = 16 mice) were anaesthetised with ketamine (100 mg/kg i.p) before decapitation. The cervical vertebral column was isolated and immersed in ice-cold oxygenated sucrose substituted artificial cerebrospinal fluid (s-ACSF) containing (in mM): 250 sucrose, 25 NaHCO_3_, 10 glucose, 2.5 KCl, 1 NaH_2_PO_4_, 1 MgCl_2_, 2.5 CaCl_2_. The spinal cord was removed and a shallow cut made down the contralateral side of the spinal cord (ventral surface) to identify the ipsilateral side for recording. Transverse slices (300 μm thick) were obtained from the C1-C2 region using a vibratome (Leica VT-1000S; Leica Microsystems, Wetzlar, Germany). All slices were transferred to an interface storage chamber containing oxygenated ACSF (118 mM NaCl substituted for sucrose in s-ACSF), and allowed to equilibrate for 1 hr at room temperature (approximately 22-24°C), prior to recording.

Slices were placed in a recording bath and continually superfused (4–6 bath volumes/min) with ACSF maintained at near-physiological temperature (33°C). Whole cell patch clamp recordings were obtained from visualised SDH neurons (lamina I-II) using infrared differential interference contrast optics and an infrared-sensitive camera (Hamamatsu C2400-79C, Hamamatsu City, Japan). Recordings were restricted to the SDH by targeting neurons within or dorsal to the substantia gelatinosa (lamina II), which appears as a translucent band in transverse spinal cord slices. Patch pipettes (2–5 MΩ) were filled with a K^+^-based internal solution containing (in mM): 135 KCH_3_SO_4_, 6 NaCl, 2 MgCl_2_, 10 HEPES, 0.1 EGTA, 2 MgATP, 0.3 NaGTP, adjusted to a pH of 7.3 with KOH. Whole cell recordings were obtained using an Axopatch 200B amplifier (Molecular Devices, Sunnyvale, CA, USA), connected to a Macintosh G4 computer via an InstruTECH ITC18 digitiser (HEKA Instruments, Bellmore, NY, USA).

The whole cell recording configuration was first established in voltage clamp (holding potential −60 mV). Series resistance (R_s_), input resistance (R_IN_), and capacitance were measured from the averaged response (5 repetitions) to a 5 mV hyperpolarising voltage step. These parameters were observed following each protocol, and data were rejected if values changed by >20%. Initially, spontaneous excitatory post-synaptic currents (sEPSCs) were recorded for each neuron in voltage-clamp (holding potential −60 mV; 60 s duration). The recording mode was then switched to current clamp. The membrane potential recorded following this switch (~15 s later) was designated resting membrane potential (RMP). A series of depolarising ‘step-current’ injections (20 pA increments; 800 ms, delivered every 8 s) were then injected in order to study action potential (AP) discharge in each recorded neuron. Finally, the recording configuration was returned to voltage clamp and a protocol was run to study voltage-gated subthreshold ionic currents. This protocol first hyperpolarised the membrane potential to −90 mV (for 1 s) before delivering a depolarising step to −40 mV (for 200 ms) before returning to −60 mV. All membrane currents were filtered at 2 kHz and stored on a Macintosh G4 computer using Axograph v.10 (Axon Instruments, Foster City, CA, USA) for future analysis.

### Data analysis

All data were analysed off-line using semi-automated procedures within Axograph software. sEPSCs were detected and captured using a scaled template method and subsequently inspected [14]. Some events were rejected if they overlapped or did not include a stable baseline before the rising phase of the captured event. For each recording, stationary plots of sEPSC interevent interval were constructed for the duration of the analysis period. These plots were inspected to ensure no time dependent change or heavy clustering of data points, which would both be indicative of burst activity, were included in the analysis. Several sEPSC parameters were measured including mean amplitude, rise time, decay time and instantaneous frequency. Individual APs evoked during step current injection were detected and captured using the derivative threshold method for spike initiation (set at dV/dt ≥ 20 Vs^−1^). The pattern of AP discharge was classified into categories using previously described criteria [15,16] as; single spiking, initial bursting, tonic firing, delayed firing, or reluctant firing. Likewise, subthreshold currents (I_Ar_, I_As_, T-type calcium, I_h_, & mixed) were classified and compared between control, sham and experimental (carrageenan-injected) animals.

### Statistical analysis

All statistical analyses were carried out using SPSS statistics software v.18.0 (SPSS Inc., Chicago, IL, USA). One-way ANOVA coupled with Student-Newman-Keuls and Scheffe post-hoc tests were used to compare variables from control, sham and experimental animals. Data with unequal variances were compared using the non-parametric Kruskal-Wallis test. Chi-square tests were utilised to determine whether the prevalence of discharge categories and subthreshold currents differed between the groups. Statistical significance was set at p < 0.05. All values are presented as the mean ± S.E.M.

## Results

**Figure 1:**
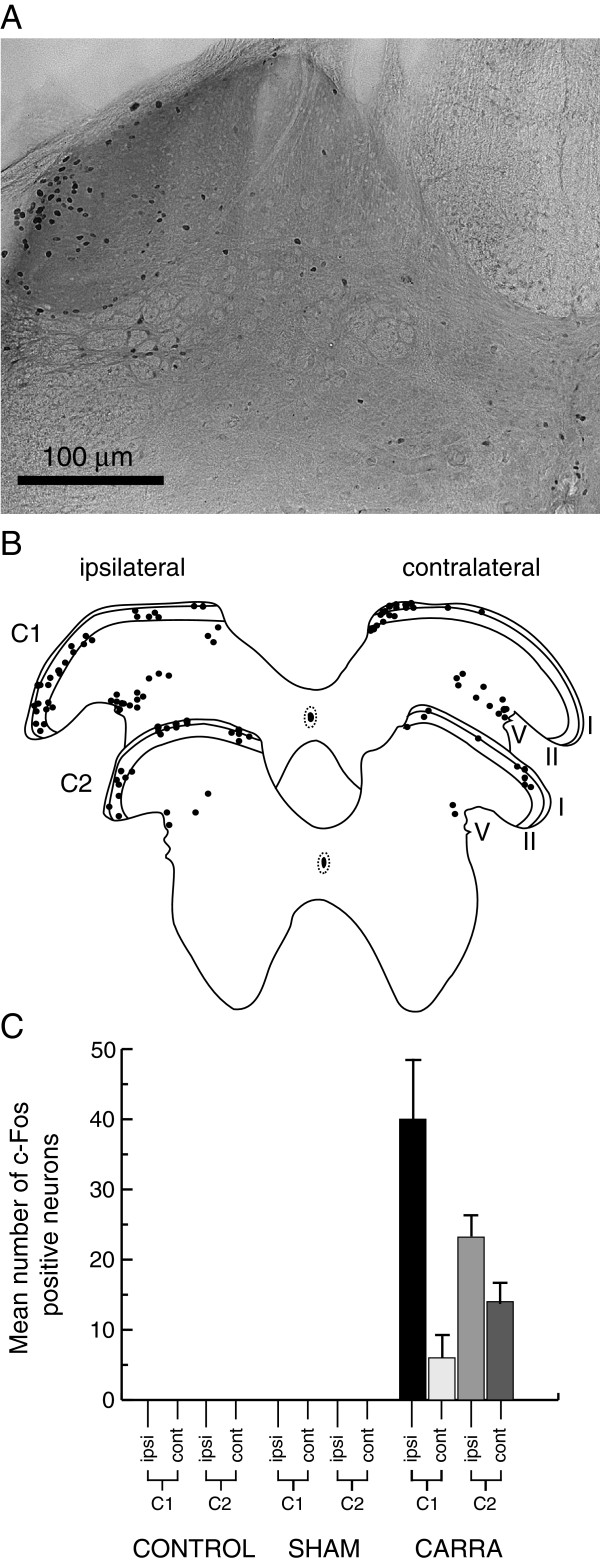
**Neck muscle inflammation activates upper-cervical dorsal horn neurons. A**. Representative image of a transverse spinal cord section (C2 level) from a carrageenan-injected animal showing cFos labelling in the ipsilateral dorsal horn. Note, labelled neurons are concentrated in the superficial dorsal horn with a lateral bias. **B**. Maps showing group data from carrageenan-injected animals. The location of cFos labelled neurons have been collapsed onto standardised C1 and C2 templates (adapted from [13]). **C**. Graph comparing mean number of cFos positive neurons in control, sham, and carrageenan-injected animals. Data are separated into ipsilateral and contralateral staining across spinal levels C1 and C2.

Experiments were designed to characterise the early events that occur in SDH neurons following acute neck muscle inflammation. To establish the segmental level and distribution of activated neurons, spinal cords were processed to visualise the immediate early gene product c-Fos (Figure 1). The level of neuronal activation (determined by number of c-Fos positive profiles) was assessed in the C1-C2 spinal segments of control, sham, and carrageenan-injected animals.

c-Fos expression remained below detectable levels in the C1-C2 spinal segments of control (un-injected; n = 4) and sham (PBS-injected; n = 3) mice. In contrast, spinal cord sections from carrageenan-injected animals contained substantial c-Fos expression in both ipsilateral and contralateral dorsal horns (Figure 1). This expression was concentrated in the SDH region (laminae I - II) in C1 and C2 spinal segments, with some scattered expression in lateral lamina V. The number of c-Fos positive neurons was much higher in the ipsilateral versus contralateral dorsal horn (C1, 40 ± 9 vs. 6 ± 4 cells/section; C2, 23 ± 4 vs. 14 ± 4 cells/section). Our analysis also showed that carrageenan injection into the RCM muscle resulted in greater neuronal activation in the C1 versus C2 spinal segment and the ipsilateral versus contralateral bias was more pronounced in C1 segments (i.e. C1 - ipsi 87% versus contra 13% of c-Fos positive cells; C2 - ipsi 62% vs. contra 38% of c-Fos positive cells). In summary, the greatest degree of neuronal activation following acute inflammation of the RCM muscle occurred in the ipsilateral SDH of the C1 spinal segment. Electrophysiological recordings were therefore obtained on the ipsilateral side of slices prepared from C1 and C2 spinal cord segments from control, sham, or carrageenan-injected animals.

**Figure 2:**
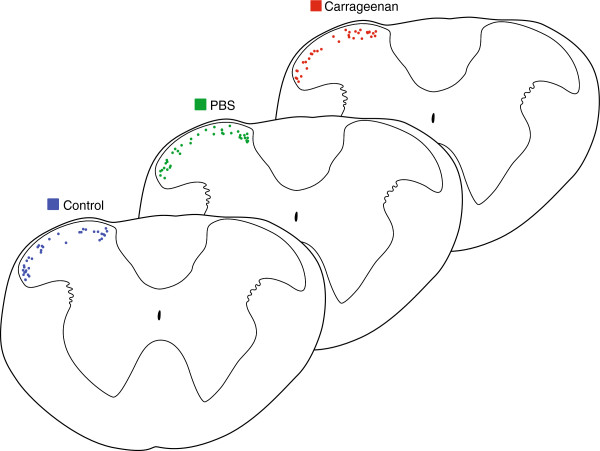
**Location of recorded neurons in spinal cord slices.** The location of recorded neurons was documented to check and account for bias. Low magnification images (5×) were captured at the conclusion of recording sessions with the recording pipette still in place. Recording locations were then plotted on a standardised transverse template of the upper cervical spinal cord. Recording locations were then consolidated onto a single template for control, sham, and carrageenan-injected experiments. Comparison of these maps indicates similar sampling in both mediolateral and dorsoventral planes for each experimental condition.

Whole cell patch-clamp recordings were obtained from 205 SDH neurons in 41 animals from carrageenan-injected (n = 58 cells from 10 mice), sham (n = 68 cells from 15 mice), and control (n = 79 cells from 16 mice) animals. These experiments examined a range of electrophysiological properties known to contribute to neuronal excitability and plasticity. The location of recorded neurons was documented in latter experiments (after image capture software) to ensure similar sampling occurred throughout the SDH under all experimental conditions. Figure 2 summarises the results of this analysis and confirms that similar regions of the upper cervical SDH were sampled in carrageenan-injected, sham and control mice (n = 33, 39, and 36 neurons, respectively).

Several passive membrane properties did not differ in neurons from carrageenan-injected, sham and control mice. These included input resistance (447.9 ± 33.1 MΩ vs. 395.1 ± 28.6 MΩ vs. 401.9 ± 23.8 MΩ, carrageenan-injected, sham and control, n = 58, 68, & 79 respectively, p = 0.42) and RMP (−59.2 ± 1.5 mV vs. −58.0 ± 1.4 mV vs. −60.7 ± 1.3 mV; p = 0.39). These results suggest acute neck muscle inflammation does not alter the passive properties of upper cervical SDH neurons.

**Figure 3:**
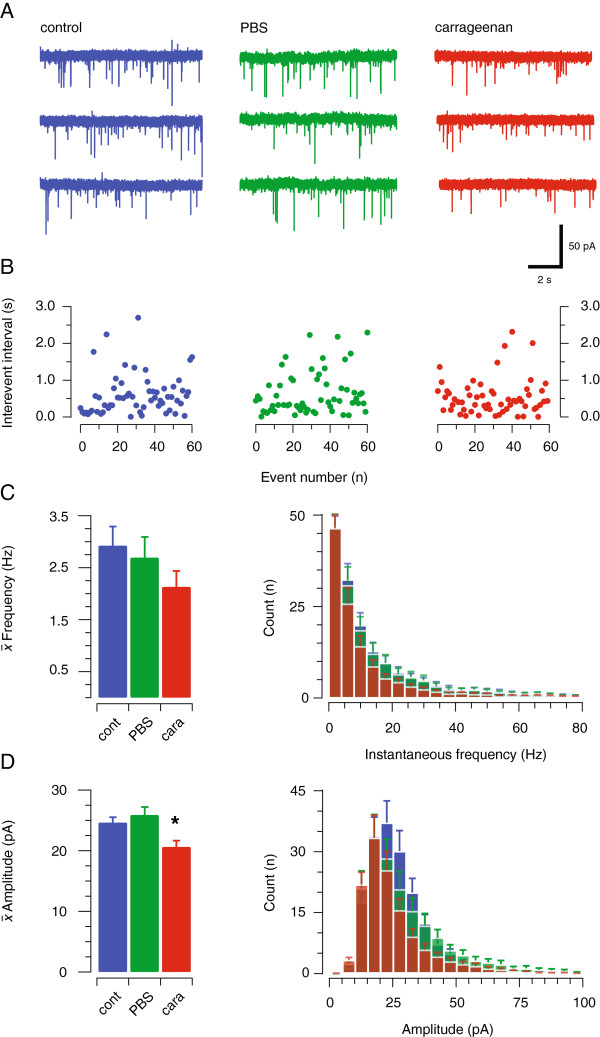
**Excitatory synaptic drive is altered in the upper cervical dorsal horn by acute neck muscle inflammation. A**. Raw data showing representative spontaneous excitatory postsynaptic currents (sEPSCs, 30 s of continuous data) in SDH neurons from control, sham, and carrageenan-injected animals. sEPSCs were blocked by CNQX (10 μM, not shown). **B**. Stationary plots of sEPSC interevent interval (IEI) for the control, sham, and carrageenan-injected recordings shown in **A**. Note that these plots do not contain any time dependent trends, or tight clustering of data points that would indicate sEPSC bursting. **C**. Group data showing mean (left) and instantaneous (right) sEPCS frequency were similar in control, sham, and carrageenan-injected animals. **D**. Group data shows sEPCS amplitude (left) was significantly reduced in carrageenan-injected animals. Overlaid average sEPSC amplitude histograms (right) show a leftward shift in the distribution for carrageenan-injected animals.

We next recorded spontaneous excitatory postsynaptic currents (sEPSCs) to determine whether acute neck muscle inflammation alters excitatory synaptic drive in spinal circuits (Figure 3). sEPSCs represent the postsynaptic response to action potential dependent and spontaneous neurotransmitter release from presynaptic terminals. Mean sEPSC frequency was similar in carrageenan-injected, sham and control animals (2.13 ± 0.31 Hz vs. 2.69 ± 0.40 Hz vs. 2.92 ± 0.37 Hz, n = 58, 68, & 79 respectively; p = 0.31) (Figure 3B). sEPSCs time course was also similar with mean values for rise time (0.65 ± 0.02 ms vs. 0.65 ± 0.02 ms vs. 0.68 ± 0.02 ms, respectively; p = 0.39) and decay time constant (2.14 ± 0.08 ms vs. 2.23 ± 0.11 ms vs. 2.33 ± 0.10 ms, respectively; p = 0.39) being similar in carrageenan-injected, sham and control animals. In contrast, mean sEPSC amplitude was reduced by ~20% in neurons from carrageenan-injected mice (20.63 ± 1.05 vs. 24.64 ± 0.91 and 25.87 ± 1.32 pA, carrageenan-injected, sham and control, respectively; p = 0.004) (Figure 3C). The combined effect of these properties would result in a ~30% charge transfer (calculated as area under curve) in carrageenan-injected animals (66.55 ± 4.02 pA.ms vs. 91.50 ± 6.06 pA.ms vs. 96.94 ± 4.17 pA.ms; carrageenan-injected, sham, and control animals, respectively; p = 0.0001). Thus, excitatory drive to SDH neurons is reduced significantly in the ipsilateral dorsal horn following acute neck muscle inflammation.

**Figure 4:**
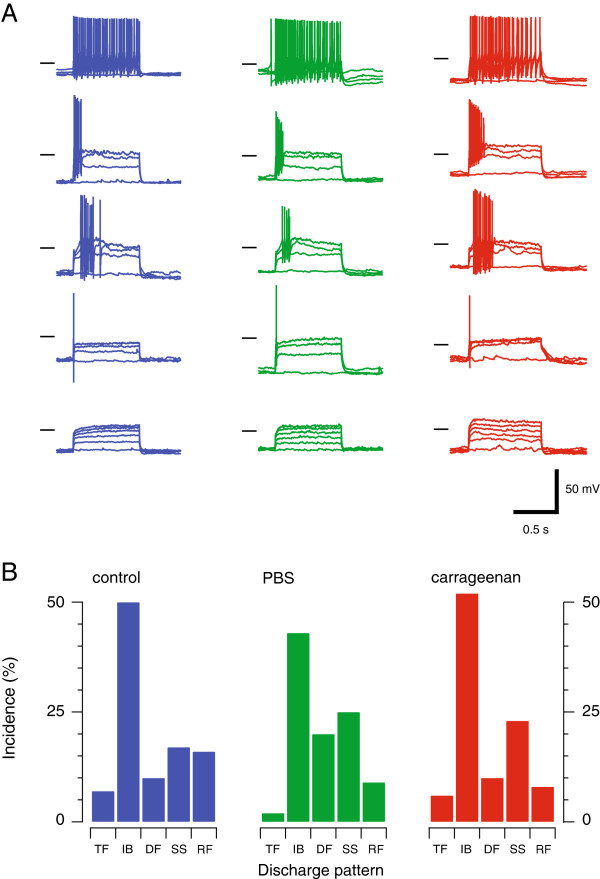
**Acute neck muscle inflammation does not alter action potential discharge properties*. *A**. Dorsal horn neurons can be classified by the temporal features of their action potential discharge. We have previously described five AP discharge patterns under in vitro [16] and in vivo conditions [15], and also across the lumbar, thoracic and cervical spinal cord in adult mice [17]. Traces show representative examples of each AP discharge pattern (Tonic Firing - TF, Initial Bursting - IB, Delayed Firing - DF, Single Spiking - SS, and Reluctant Firing RF) recorded from control (left), sham (middle), and carrageenan-injected animals (right). Horizontal bars denote −50 mV membrane potential. **B**. The incidence of the five AP discharge patterns was similar in control, sham, and carrageenan-injected animals.

In a subset of recordings (n = 48, 44, 58; control, sham and carrageenan-injected, respectively) we examined AP discharge, evoked by step current injection, to assess whether acute neck muscle inflammation altered the intrinsic properties (and excitability) of upper cervical SDH neurons. The features of voltage responses exhibited at multiple current step injections were used to classify AP discharge patterns into five categories, as previously described [15,16]. Single spiking neurons, discharged one AP at the beginning of step current injection regardless of current intensity; initial bursting neurons discharged a brief, adapting train of APs limited to the beginning of the current step; tonic firing neurons exhibited sustained discharge for the duration of the current step; delayed firing neurons discharged APs after a delay from step current onset; and reluctant firing neurons failed to fire APs in response to any current step, independent of intensity. All discharge patterns were observed under the three experimental conditions (Figure 4A), and there was no significant difference in the proportion of these discharge categories in control, sham and carrageenan-injected animals (Figure 4B, *χ*^2^; p = 0.27).

**Figure 5:**
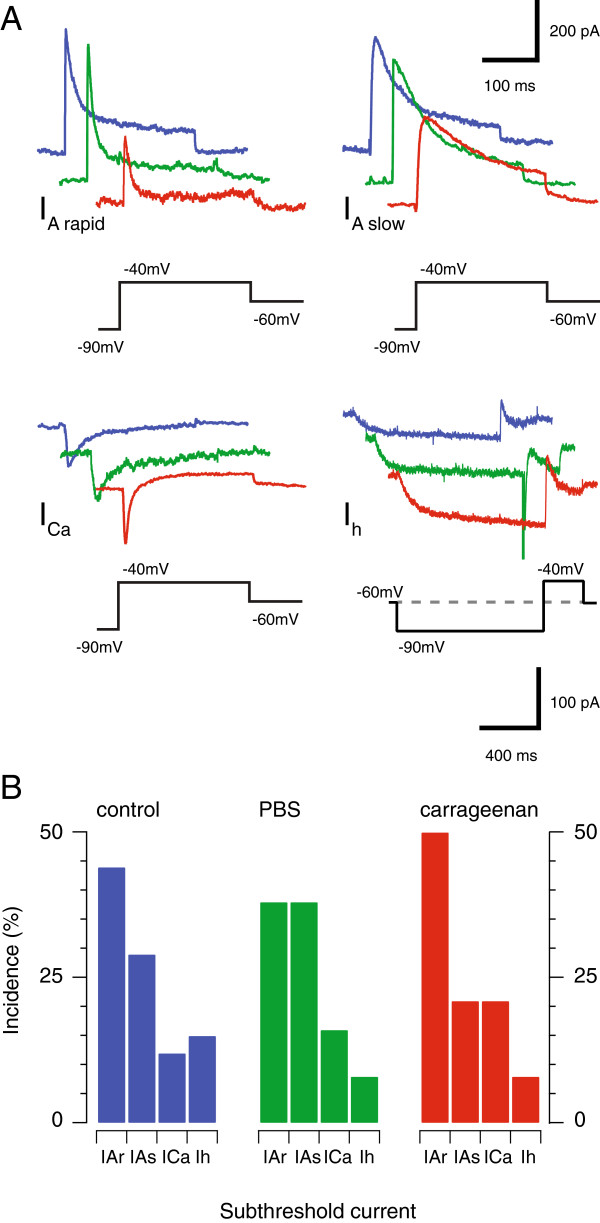
**Acute neck muscle inflammation alters subthreshold voltage-activated currents. A**. Traces show representative voltage-activated current recordings (from control, sham, and carrageenan-injected animals) in response to voltage step protocol (bottom black traces, note different scale for lower right I_h_ currents). We have previously shown this protocol identifies one of four dominant currents in dorsal horn neurons: rapid A-type potassium currents (I_A rapid_); slow A-type potassium currents (I_A slow_); T-type calcium currents (I_Ca_); and a hyperpolarisation-activated non-selective cation current (I_h_). Analysis of these currents across the three experimental conditions showed the characteristics of I_A slow_, I_Ca_, or I_h_ did not differ under the three conditions. In contrast, the amplitude of the I_A rapid_ current was reduced in carrageenan-injected animals (see text). **B**. Plots show the incidence of sub-threshold voltage activated current types was similar in control, sham, and carrageenan-injected animals.

In some neurons we also tested for the presence of the four main subthreshold currents that shape AP discharge in SDH neurons (n = 38, 39, 41; carrageenan-injected, sham, and control, respectively, Figure 5). This analysis employed a voltage-step protocol to identify different subthreshold currents in adult mouse SDH neurons [18]. These include two types of outward potassium current that are activated by a depolarising step (−90 to −40 mV). The first displays fast activation and inactivation kinetics and is termed a rapid A-type current (I_Ar_), while a second exhibits slower activation/inactivation kinetics and is termed a slow A-type current (I_As_). These two currents are easily differentiated as the rapid form (I_Ar_) was fully inactivated during a 200 ms activation step (to −90 to −40 mV), whereas the slower form did not fully inactivate over the same period. A criterion threshold of 50 ms for the outward current inactivation time constant was used to classify currents as either I_Ar_ (<50 ms) or I_As_ (>50 ms). This approach resulted in two non-overlapping distributions in recordings from control, sham and carrageenan-injected mice. The other major subthreshold current types are inward currents. The first is an inward current with rapid activation/inactivation kinetics (in response to a −90 mV to −40 mV step), consistent with the low threshold ‘T-type calcium current’ (I_Ca_; Figure 5A). The second, inward current is activated by hyperpolarisation (−60 mV to −90 mV step) and exhibits slow activation kinetics consistent with the well-described current termed I_h_ (Figure 5A). Importantly, coexpression of the currents observed using our protocol was not frequently observed. For example, a combination of I_Ca_ and I_h_ occurred in <10% of the neurons sampled (n = 4/41, 2/39, and 2/38, control, sham and carrageenan-injected respectively). Co-expression of other currents was not directly assessed in these experiments, as this would have required pharmacological analysis, and limited the collection of data that was the principal focus of our experiments. Previous work using pharmacological analysis, albeit in lumbar SDH neurons, suggests a single subthreshold current typically dominates in response to our protocol [18]. We do not discount the possibility, however, that combinations of I_Ar_, I_As_, and I_Ca_ may exist in some of these neurons or that the incidence of mixed currents is higher in upper cervical SDH neurons.

Each of the four subthreshold currents was present in neurons from control, sham, and carrageenan-injected animals (Figure 5B). Under each condition, I_Ar_ and I_As_ were the dominant currents, while I_Ca_ and I_h_ was observed in < 30% of neurons. Collectively, the relative proportion of each subthreshold current did not differ across groups (Figure 5B, *χ*^2^; p = 0.27). Further analysis showed the properties of I_As_, I_Ca_ and I_h_ were similar under control, sham and carrageenan-injection conditions. Specifically, the mean amplitude of I_As_ (606 ± 309 pA vs. 617 ± 107 pA vs. 475 ± 113 pA, for carrageenan-injected, sham and control; n = 4, 6, & 5 respectively, p = 0.81) and mean inactivation time constant (130.1 ± 15.5 ms vs. 140.9 ± 14.1 ms vs. 181.0 ± 38.2 ms, respectively; p = 0.30) were similar across experimental conditions. Mean amplitude of I_Ca_ (153 ± 43 pA vs. 273 ± 137 pA vs. 194 ± 80 pA, for carrageenan-injected, sham and control; n = 7, 5, & 8 respectively, p = 0.61) and mean inactivation time constant (37.0 ± 13.4 ms vs. 39.3 ± 6.9 ms vs. 31.6 ± 11.0 ms, respectively; p = 0.90) did not differ. Finally, the amplitude (79.1 ± 7.2 pA vs. 52.3 ± 5.3 pA vs. 50.6 ± 21.7 pA, for carrageenan-injected, sham and control; n = 3, 3, & 7 respectively, p = 0.66) and mean activation time constant (112.4 ± 17.5 ms vs. 108.7 ± 55.8 ms vs. 149.7 ± 31.0 ms, respectively; p = 0.68) of I_h_ were similar under each condition.

In contrast, several properties of I_Ar_ were altered in the carrageenan-injected animals. The amplitude (238 ± 33 vs. 593 ± 167 pA vs. 494 ± 96 for carrageenan-injected, sham and control, n = 19, 14, & 18 respectively, p = 0.045) and inactivation time constant (12.9 ± 1.5 vs. 15.3 ± 1.4 ms vs. 22.1 ± 3.6, respectively; p = 0.031) were both reduced. Together, these results suggest the effect of I_Ar_, the dominant subthreshold current in upper cervical SDH neurons (Figure 5B), is reduced in following acute neck muscle inflammation.

## Discussion

We assessed the impact of two hours of acute neck muscle inflammation on the properties of SDH neurons in the upper cervical spinal cord. Our c-Fos experiments showed carrageenan-induced muscle inflammation produced neuronal activation in both ipsilateral and contralateral laminae I – II, and to a lesser extent in lamina V of the C1 and C2 spinal segments. These findings confirmed that neurons in laminae I - II should be the principal target in subsequent electrophysiological recordings from acutely prepared spinal cord slices.

Assessment of c-Fos expression, the protein product of the proto-oncogene *c-fos*, is a well-established method for determining neuronal activation after noxious peripheral stimulation [19,20]. In our study, c-Fos expression occurred in neurons located predominantly in laminae I - II. This matches the terminal field distribution of nociceptive afferent fibres [2], and the location of c-Fos expressing neurons after application of noxious peripheral stimuli [20]. Some c-Fos labelling was also observed in deeper layers (lateral lamina V) of the dorsal horn. These superficial and deep locations closely match those noted in other rodent injury models involving deep tissues, such as muscle. For example in rat, chronic constriction injury of the sciatic nerve [21], adjuvant-induced arthritis [22], and nerve growth factor injection into semispinal neck muscles [23] resulted in c-Fos labelling in laminae I-II and V-VII. In contrast, capsaicin injection into the trapezius and splenius muscles of cats does not label many neurons in lamina II [24]. Thus, some differences exist in the extent to which neurons in laminae II are involved in processing noxious input from muscle across species.

No labelling was observed in control or sham (saline injection) animals in our c-Fos experiments. These data suggest neither handling nor the manipulation of muscle tissue associated with the injection protocol resulted in significant activation of dorsal horn interneurons. Similar results have been observed for limb muscles when c-Fos expression has been compared with that in vehicle-injected animals [25]. Our unilateral carrageenan injections resulted in c-Fos labelling in both ipsilateral and contralateral superficial laminae (I-II) with some labelling in deeper laminae. Bilateral c-Fos expression has been observed after capsaicin injection into other neck muscles (trapezius and splenius capitis) in cats [24]. This contrasts with the data for limb muscles where inflammation produces neuronal activation that is confined to the ipsilateral side of the spinal cord. Thus, the major pattern of c-Fos activation we described following carrageenan injection into RCM, fits with activation of muscle nociceptive pathways in axial musculature. Thus, recording from neurons in ipsilateral laminae I-II should detect inflammation-related changes in the spinal dorsal horn.

In our electrophysiological analysis of SDH neurons in spinal cord slices prepared two hours after RCM inflammation we observed two major changes: i) AMPA-mediated excitatory drive decreased and ii) the amplitude and kinetics of the rapid, but not slow I_A_ type potassium current were altered. Assuming acute neck muscle inflammation enhanced nociceptive signaling (cf*.* our c-Fos results above), it is surprising that we found excitatory drive to SDH neurons was reduced (as assessed by sEPSC amplitude and charge). It is noteworthy, however, that other studies have also described reduced excitatory drive to SDH neurons in pain models, specifically in GAD67 positive inhibitory interneurons [26]. In these experiments the frequency of spontaneous excitatory input, rather than amplitude, was reduced in neuropathic animals. The authors proposed that reduced excitatory drive to inhibitory interneurons, when placed in the context of nociceptive signaling, would reduce inhibitory drive in the SDH and contribute to hyperalgesia. While our data does not allow us to identify recorded neurons as excitatory or inhibitory, similar plasticity in the form of reduced sEPSCs onto inhibitory interneurons would contribute to enhanced nociceptive signaling in acute neck muscle inflammation.

Regardless of the identity of neurons that undergo reduced excitatory drive in our model, numerous studies have confirmed that inflammation alters the expression of AMPA type glutamate receptors. For example, expression of calcium permeable AMPA receptors containing GluR1 subunits has been shown to increase, whereas expression of calcium impermeable GluR2 containing subunits was reduced [27-31]. Importantly, all these studies assessed receptor expression 24 hours after peripheral inflammation. In contrast, our data showing sEPSC kinetics were unaltered in carrageenan-injected recordings imply that AMPA receptor expression is unchanged at the two-hour time point used following axial muscle inflammation. Phosphorylation of GluR1 and GluR2 has, however, been demonstrated at time points commensurate with those in our study [32]. This would enhance excitatory drive in SDH neurons. Importantly, most GluR1/2 subunit expression and phosphorylation studies have used biomolecular techniques in spinal cord homogenates where synaptic and extrasynaptic receptors and the precise laminae location of neurons are not known. Thus our experiments, which assessed synaptic receptor function in specific laminae, suggest levels of GluR plasticity vary according to time after inflammatory insult.

Several electrophysiological studies have examined the properties of primary afferent synapses in the SDH after peripheral inflammation, and report that primary afferent synaptic function was generally enhanced [33,34]. Again this contrasts with the reduced excitatory drive that we observed. However, our sEPSCs recordings will have included excitatory currents arising from local interneurons, descending systems, as well as primary afferents. Relevant to this point, previous work has shown that ablation of afferent input by dorsal rhizotomy, or selective removal of peptidergic afferents with neonatal capsacin treatment, does not reduce mEPSC frequency recorded from SDH neurons in spinal slices [35,36]. This suggests that the majority of sEPSCs recorded in an isolated spinal slice such as that in our experiments, come from local interneurons and not primary afferent terminals. Thus, any change to primary afferent synapses may have gone undetected in our experiments.

When sEPSCs have been recorded in the SDH in peripheral inflammation models it appears their properties are not altered within the first 24 hours after inflammation [37,38]. Interestingly, the Li et al. study showed that sEPSC frequency was enhanced when inflammatory insults were delivered to neonates but not in animals older than P14. The most likely explanation for the reduced sEPSC amplitudes we observed comes from work that has assessed the contribution of the initial primary afferent barrage and its time course during an inflammatory insult. For example, peripheral nerve block with lignocaine during muscle inflammation prevents the development of plasticity (or central sensitisation) in spinal neurons, whereas nerve block outside the first two hours of inflammation does not protect against central sensitisation [39]. This finding suggests primary afferent input within the first two hours of inflammation plays a crucial role in establishing central sensitisation. In our experiments, the barrage of primary afferent input would be expected to be the principle driver of central sensitisation, which is reflected as the expression of c-Fos in certain neuronal populations. The fact, however, that we did not observe an enhancement of sEPSC frequency or amplitude may partly be explained by the in vitro slice preparation we used. Specifically, previous work has shown that the removal of populations of primary afferent inputs either by dorsal rhizotomy or neonatal capsacin does not affect the properties of sEPSCs recorded in slices [35,38]. This implies that most sEPSCs recorded in slices originate from local interneurons rather than primary afferents. Further support of our interpretation comes from work in an in vitro hemisected spinal cord preparation where inflammation-induced central sensitisation was only detected six hours after inflammation in the absence of peripheral input [34]. These data, when combined with our finding that excitatory drive is decreased two hours after acute muscle inflammation, suggests a complex sequence of events occurs over the onset, establishment and maintenance of inflammatory pain and associated central sensitisation.

The reduced excitatory drive we observed two hours after acute neck muscle activation was accompanied by a reduction in the amplitude and time course of the I_Ar_ - type potassium current. I_Ar_ currents are expressed widely in the CNS and regulate AP discharge and neuronal output. In the dorsal horn, I_Ar_ currents are thought to play an important role in reducing neuronal excitability. For example, I_Ar_ currents are preferentially associated with the delayed firing pattern of AP discharge (Figure 4A). Likewise, SDH neurons expressing delayed firing and I_Ar_ currents exhibit reduced AP discharge compared to tonic firing and initial bursting neurons when activated by current protocols they are likely to receive in vivo (synaptic vs. square step current injection) [40]. Furthermore, a number of studies have suggested I_Ar_ expression is a feature of excitatory interneurons [41,42]. This suggests that diminished I_Ar_ would substantially increase excitability and nociceptive signaling in the dorsal horn. Thus, any intervention that reduces I_Ar_ current in SDH neurons leads to enhanced excitability.

Comparable modulation of I_Ar_ in the dorsal horn has been reported after peripheral inflammation. For example, enhanced excitability in dorsal horn neurons is observed as early as one hour after carrageenan injection into the hindpaw of young (P7-12) mice [43]. A detailed analysis of I_Ar_ potassium currents in these animals identified a shift in the current’s steady state inactivation. The authors suggested this would act like “a relaxation of the brake on excitability at physiologically meaningful potentials”. Interestingly, we have previously demonstrated the converse (i.e., enhanced braking) in spastic mice with glycine receptor mutations [18]. In this case, modulation of I_Ar_ was seen as a compensatory mechanism to enhance neuronal inhibition and maintain stable sensory processing. Together, this work suggests I_Ar_ can be strongly modulated to maintain an excitability set-point in the SDH. Importantly, a signalling pathway involving metabotropic glutamate receptor activation and extracellular signal-regulated kinase dependent phosphorylation of Kv4.2 channels has been shown to regulate I_Ar_ currents in SDH neurons [44,45]. Furthermore, this work demonstrated that inflammation of peripheral tissues can activate this pathway and reduce I_Ar_. This provides a potential mechanism for our observations during acute neck muscle inflammation.

Given I_Ar_ currents are diminished in SDH neurons from carrageenan-injected animals one might expect this to impact on neurons with the delayed- and reluctant firing AP discharge pattern as the two have been associated in a number of previous studies [18,46,47]. For example, diminished I_Ar_ would either reduce the delay before AP discharge or reduce the proportion of neurons exhibiting delayed and reluctant firing and convert them to more excitable forms of discharge such as tonic firing or initial bursting. There was, however, no difference in the incidence of AP discharge patterns across the three conditions: control, sham and carrageenan-injected. This finding may be explained, at least partly, by the biophysical properties of I_Ar_ currents, which are inactivated at depolarised membrane potentials [16,47]. The membrane potential of neurons in our recordings was approximately −60 mV across all three conditions. At this potential much of I_Ar_ (~80 - 90%) is inactivated and therefore not able to reduce or delay AP discharge. Under in vivo conditions, however, when membrane potential is fluctuating due to the combination of inhibitory and excitatory synaptic input, I_Ar_ currents could have a greater impact on neuronal output. Thus, under in vivo conditions the reduction in I_Ar_ we observed could enhance excitation and disrupt normal sensory processing.

Ultimately, our findings must be placed in the context of the whole animal. Fortunately, relevant data are available on the time course of pain related behaviours following carrageenan injections. Most of this work has involved carrageenan injection into the plantar surface of the hindpaw [48-52] (rather than muscle). Carrageenan causes rapid edema, which peaks 3–4 hours post injection and largely resolves by 24–72 hours [53]. Behaviourally, carrageenan’s capacity to induce thermal and mechanical hyperalgesia is highly reproducible. These indices of altered pain sensation have been detected as early as 15 minutes after carrageenan injection [48], though typically studies report effects to be fully developed at 1–4 hours [52]. Following onset, some variability exists in duration of the carrageenan-induced mechanical and thermal hyperalgeisa. This variability might reflect the carrageenan concentration (typically 1-3% is injected) as well as the species studied. However all work suggests mechanical and thermal hyperalgeisa persists for at least 24 hours [49]. The few studies that have injected carrageenan into muscle also support a rapid onset for mechanical and thermal hyperalgesia, which is fully developed by two hours and can last as long as 4–8 weeks [54,55]. Thus our study has described a number of central changes to both the synaptic and intrinsic membrane properties of SDH neurons that equate to a relatively early time point in the carrageenan-induced inflammatory pain model.

In summary, the present study suggests two hours of acute neck muscle inflammation represents a transition point between the involvements of peripheral and/or central sensitisation. As our study was undertaken at one time point post inflammation it is difficult to predict where each of the alterations we observed lies in terms of the train of events that leads to central sensitisation. The surprising result that sEPSC amplitude and charge are reduced, suggest some excitatory inputs are depressed in the hours following inflammation. This may reduce local excitatory drive in the face of the initial excitatory barrage arising from the periphery. Alternatively, if the neurons that experience a reduction in excitatory drive were inhibitory, an associated reduction in the activity of this population would reduce inhibition in the SDH and contribute to enhanced nociceptive signaling. It will be important in future studies to assess whether our observation persists after peripheral drive abates following the resolution of inflammation in the periphery. The diminished I_Ar_-current after two hours of acute inflammation is, however, consistent with inflammation-induced hyperexcitability in the spinal cord dorsal horn. Furthermore, there is a clear signalling pathway that links hyperexcitation (and neuronal depolarisation), with ERK kinase-dependent down regulation of A-currents [56]. Thus, the early barrage of afferent input from inflamed muscle could underpin this observation. Our findings when combined with the existing literature on altered spinal cord processing, albeit at longer times after acute insult, suggest a complex sequence of events occurs in the dorsal horn following neck muscle inflammation. A greater understanding of the order and duration of these changes may help to uncover new strategies for blocking the transition from peripheral to central sensitisation.

## Abbreviations

AP: Action potential; PB: Phosphate buffer; PBS: Phosphate-buffered saline; RCM: Rectus capitis major; sEPSC: Spontaneous excitatory post synaptic current; hrs: Hours.

## Competing interests

The authors declare that they have no competing interests.

## Authors’ contributions

BMH carried out all surgery and electrophysiological recording, and helped draft the manuscript. DIH carried out c-Fos staining. BMH and BAG analysed the electrophysiological and anatomical datasets. BAG, PSB and RJC conceived the study, participated in its coordination and drafted the final version of the manuscript. All authors have read and approved the final version of the manuscript.
